# Nurses’ turnover intention, hope and career identity: the mediating role of job satisfaction

**DOI:** 10.1186/s12912-022-00821-5

**Published:** 2022-02-10

**Authors:** Huiling Hu, Chongkun Wang, Yue Lan, Xue Wu

**Affiliations:** School of Nursing, Peking University, Beijing, 100191 P.R. China

**Keywords:** Personnel turnover, Hope, Career identity, Job satisfaction, Nurses, Mediating effect

## Abstract

**Background:**

A high turnover rate has become a critical issue in the field of nursing and how to tackle the problem of nursing turnover has received increased attention worldwide. Hope, career identity, job satisfaction may be useful for reducing turnover. The aim of this study is to explore the relationships among career identity, hope, job satisfaction, and the turnover intention of nurses, and to test the mediating role of job satisfaction on the associations of hope and career identity with turnover intention.

**Methods:**

A descriptive cross-sectional design was used. A total of 500 nurses were recruited from five comprehensive tertiary hospitals using convenience sampling. The questionnaire included items about sociodemographic information as well as the Adult Dispositional Hope Scale, Nursing Career Identity Scale, Job Satisfaction Index Scale, and Nurse Turnover Intention Scale. Pearson’s correlation, multiple linear regression, and structural equation modeling were used to analyze the data. We describe the study in accordance with the STROBE statement.

**Results:**

Hope (*r* = − 0.227, *p* < 0.001) and career identity (*r* = − 0.342, *p* < 0.001) were negatively correlated with turnover intention. Job satisfaction played a completely mediating role on the associations of hope and career identity with turnover intention (β_1_ = − 0.09, β_2_ = − 0.33).

**Conclusions:**

Job satisfaction mediated the associations of career identity and hope with turnover intention. Thus, effective measures can be taken to enhance nurses’ hope and career identity in order to improve their job satisfaction and thereby reduce their turnover intention. Providing nurses with more support, helping them find a spiritual foundation, and holding mindful activities that stimulate positive emotions are helpful. In addition, colleges should pay more attention to instilling nursing students with career identity and nursing values.

## Background

Nursing shortages have become a critical issue for healthcare systems all over the world [1–3]. Pressure on the healthcare system keeps growing because of the aging population and the increasing need of health services, which have greatly increased the demand for nurses [3–7]. When the “Healthy China 2030” initiative was implemented in China, there was an adequate reserve of nursing human resources to meet increasing demand. However, as the WHO reported, rising demand has exacerbated a global nursing shortage, which is predicted to exceed 7.6 million by 2030 [8]. Thus, the high turnover rate of nurses is a global concern [2, 5, 9, 10]. Given the situation, the shortage of nurses will become more prominent. Though recruiting more nurses may help, retaining nurses in hospitals is another and potentially more effective method to cope with the problem of nursing shortages. Therefore, how to tackle the problem of nursing turnover has received increased worldwide attention [11, 12].

A high turnover rate has become a critical issue in the field of nursing. Intention to leave is an early indicator of actual turnover [4, 13], and one way to retain nurses is to reduce their turnover intention [1]. Turnover intention has been defined as “the last in a sequence of withdrawal cognitions, a set to which thinking of quitting and intent to search for alternative employment also belong” [14]. In simple terms, turnover intention indicates the intention to leave the job in a period ahead [15]. Turnover intention has been widely proved to be an important and practical antecedent variable of turnover, and it is the best predictor of actual turnover behavior [16]. Therefore, it is very important to identify the antecedents of turnover intention. A recent study reported that 22% of hospital nurses intended to leave their profession in less than a year because of workplace stress [17], and an earlier study reported that 15.5% of nurses intended to leave their profession [18]. Another study found 35–60% of nurses left their first place of employment within 1 year [19]. This can cause a shortage of clinical nurses, reduce nursing quality [9, 17], and increase re-recruitment costs [3]. A lower rate of nurse turnover is associated with safe and positive outcomes for patients [1]. Thus, it is critical to identity the key factors that influence and mediate nurses’ turnover intention and to develop retention strategies tailored to nurses [7].

Turnover intention is affected by career identity, and a higher level of career identity usually indicates lower turnover intention among nurses [10, 18]. Nurses’ career identity can affect nurses’ work enthusiasm and job satisfaction, so as to affect the quality of nursing [20, 21]. Career identity is a relatively abstract concept, which refers to an individual’s understanding of the social impact of his or her profession and the significance of his or her work [22], and involves the internalization of core values and perspectives [23]. It can be characterized by feelings, values, and attitudes [24]. Nurses’ career identity is positively associated with job satisfaction [25]. When nurses positively identify with their careers, the dissatisfaction created by their work environment can be suppressed to a certain extent [26]. A study of 1312 hotel employees in China found that career identity has a positive impact on employees’ job satisfaction and a negative impact on turnover intention [27]. It further explains the mediating role of job satisfaction between career identity and turnover intention. However, in the nurse group, this intermediary relationship has not been explored.

A study of 416 employees shows that there is a significant negative relationship between psychological capital and turnover intention [28]. Ayijiamali et al. and Zou et al. concluded that employees with good psychological capital will look positively at things in the organization, which largely depends on hope and optimism. More studies show that psychological capital has a direct and indirect impact on turnover intention through intermediary variables [29, 30]. Hope belongs to a kind of psychological capital and has different meanings in different situations. Hope is a positive motivation state based on the inner sense of success. It includes will power, a kind of energy and path of goal orientation, that is, the way and plan to achieve the goal [31]. Scholars have shown that hope is particularly important in the face of the intense competition and uncertainty that characterize the present work and career environment [32]. A high level of hope helps nurses appropriately deal with psychological distress and cope with difficulties at work more positively, and it can motivate their pursuit of professional development. Thus, the negative impact of dissatisfaction with the job that generates intention to leave may be diminished. It means that their job satisfaction will be higher. Hope can lead to positive arousal and persistence in pursuit of one’s goals (i.e., “energized to” motivation), which involves agency thinking, the confidence that one is capable of reaching one’s goals (“can do” motivation), as well as pathway thinking, which are a critical psychological resource related to positive human development in various life domains, including vocational pursuits [32]. A study of workers in China found that hope was negatively correlated with turnover intention [33]. Although hope is increasingly recognized to be an important psychological resource for professional development, most studies have focused on the relationship between career and psychological capital, which combines hope with optimism, self-efficacy, and resilience into a somewhat ambiguous concept, and studies on the effects of hope on job satisfaction and turnover intention are rare.

Job satisfaction has a negative association with turnover intention [2, 34], which suggests that improving the job satisfaction of nurses could be an important strategy to retain them. The results of testing the turnover model show that job satisfaction is a significant antecedent of turnover intention. In other words, when employees’ job satisfaction is low, they will increase the behavior indicating their intention to leave the organization [35, 36]. Job satisfaction is usually defined as a positive and pleasurable emotional reaction generated by an individual’s overall assessment [2]. Job satisfaction was first proposed by Hoppock et al. [37]. He believes that job satisfaction refers to an individual’s psychological and physiological satisfaction with the work itself and the environment, and it is also an individual’s subjective response to the work environment. Job satisfaction is an important part of organizational psychology and organizational behavior. Scanlan and Hazelton proposed that job satisfaction plays an intermediary role between career identity and employee turnover intention [25]. They believe that career identity enables employees to effectively obtain the required work resources, helps to create a comfortable working atmosphere, so as to improve employees’ job satisfaction and reduce employees’ turnover intention. It can be seen that career identity is the key factor affecting employees’ job satisfaction. Therefore, we propose that job satisfaction can be used as an intermediary to explain its intermediary role between career identity and turnover intention behavior. Using job satisfaction as an intermediary can deepen our understanding of how career identity affects hotel employees’ turnover intention. In addition, hope has been found to have a positive association with job satisfaction [38]. As career identity is closely related to self-efficacy, it may change attitudes towards work [39], so we can speculate that it may improve job satisfaction.

**Fig. 1:**
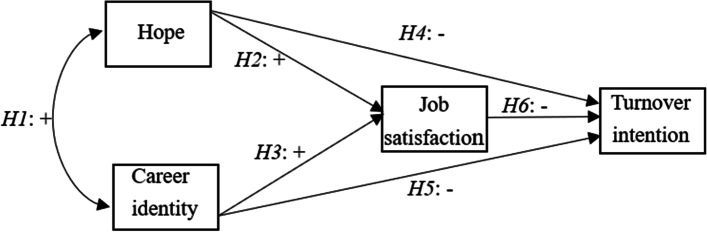
Hypothesized model of relationship between hope, career identity, job satisfaction and turnover intention

To sum up, the relationship between hope, career identity and turnover intention has been established in the literature. The research on the mediating role of job satisfaction in the relationship between hope, career identity and turnover intention is still very limited. Therefore, by combining the mediating role of job satisfaction, this study contributes to the literature on the relationship between hope, career identity and turnover intention. To our knowledge, there is no empirical study integrating these four variables into a model. We assumed that hope and career identity were negatively correlated with turnover intention, and that job satisfaction and turnover intention were negatively correlated. We hypothesized that job satisfaction plays a mediating role on the associations of hope and career identity with turnover intention. The hypothetical model we developed is shown in Fig. 1.

The purpose of this study was: (i) to investigate the turnover intention of nurses; (ii) to explore the relationships among career identity, hope, job satisfaction, and turnover intention; and (iii) to test the mediating effect of job satisfaction on turnover intention.

## Methods

### Design and data collection

This was a correlational study of 500 nurses who were recruited from comprehensive tertiary hospitals in Beijing using convenience sampling in June 2019. First, according to the convenience of sampling, we selected five comprehensive tertiary hospitals in Beijing, China. Then the nurses from all departments of the hospitals who were on duty on the survey day and were willing to cooperate with the survey were recruited. The inclusion criteria were as follows: (1) engaged in clinical work for more than 1 year; and (2) provided informed consent and volunteered to complete the questionnaires. Nurses studying in these hospitals were excluded. Trained volunteers handed out the questionnaires to potential participants, explained the purpose and principles of the study and the directions for completing the questionnaires, and obtained their informed consent. We received 493 valid questionnaires, for an effective completion rate of 98.6%. Klien suggests that a sample size of 200 or more is required to yield stable estimates for testing structural equation models (SEM) [40]. According to 10 times the number of the scale items, the sample size is 495. Considering a non-response rate of 10%, a total of 500 nurses is enough. The participants were informed that their participation was completely voluntary and anonymous. We describe the research process of the study in accordance with the Strengthening the Reporting of Observational studies in Epidemiology (STROBE) statement [41].

### Measures

#### Sociodemographic characteristics

The sociodemographic variables included gender, age, marital status, level of education, years of work, professional title, management position, and department.

#### Hope

Hope was measured with the Adult Dispositional Hope Scale (ADHS), which was developed by Snyder et al. [42] and revised by Chen, Shen, and Li [43]. The ADHS measures three dimensions of hope: agency thinking (4 items), pathway thinking (4 items), and distraction (4 items). Agency thinking refers to a group of self-belief systems that initiate individual actions and support individuals to move forward toward their goals and along the established path. Path thinking is a group of beliefs and perceptions about one’s ability to find effective ways to achieve desired goals [31]. The four questions of “distraction” are used to distract the subjects. Each item is rated from 1 to 4, except the distraction items, which are not rated. Higher scores indicate higher levels of hope. The Cronbach’s α coefficients were 0.78 for the total score, 0.73 for agency thoughts, and 0.75 for pathway thoughts. And confirmatory structural analysis showed that it had good structural validity [43].

#### Career identity

The Nursing Career Identity Scale (NCIS) was developed by scholars at Tokyo University in Japan, and it has been widely used in Japan, Sweden, and other countries. Its cross-cultural adaptation and tests of its validity and reliability were conducted by Chinese scholars [44]. The 21 items of the NCIS measure seven dimensions, including sense of grasp (3 items), sense of consistency (4 items), sense of significance (3 items), sense of self efficacy (3 items), sense of self decision (3 items), sense of organizational influence (2 items), and sense of individual influence (3 items). All the items are rated on a 7-point scale, ranging from 1 (extremely not conform) to 7 (extremely conform). The content validity index of each item is greater than 0.80 (Mean = 0.92). The Cronbach’s α of the entire scale is 0.84 and the α for the sense of organizational influence dimension is 0.69; the α for the other dimensions are equal or greater than 0.70.

#### Job satisfaction

We measured job satisfaction with the Job Satisfaction Index described by Schreisheim and Tsui at the Western Management Institute Conference in 1980 [45]. The 6 items of it measure three dimensions, including general satisfaction (2 items), intrinsic satisfaction (2 items) and extrinsic satisfaction (2 items). Its six items are rated on a 5-point Likert scale, ranging from 1 (strongly disagree) to 5 (strongly agree), with a higher total score indicating higher job satisfaction. The Cronbach’s α of the scale is 0.73. In this study, the Cronbach’s α of the scale is 0.83 and the α of dimensions are 0.78, 0.74 and 0.75 respectively.

#### Turnover intention

We used the Nurse Turnover Intention Scale (NTIS) developed by Michael and Spector [46], which contains six items that measure three dimensions: possibility of resigning from present job (2 items); motivation to seek another job (2 items); and possibility of obtaining an external job (2 items). All the items were rated on a 4-point scale: never = 1, seldom = 2, occasionally = 3, and often = 4. The total average score ≤ 1 indicates very low turnover intention, > 1 and ≤ 2 indicate low turnover intention, > 2 and ≤ 3 indicate high turnover intention, and > 3 indicate very high turnover intention. The Cronbach’s α of the scale is 0.77 and the content validity index is 0.68. In this study, the Cronbach’s α of the scale is 0.83 and the α of dimensions are 0.79, 0.63 and 0.72 respectively.

### Data analysis

First, descriptive statistics were calculated for the sociodemographic characteristics, which are presented as frequency counts and percentages, and the main study variables (i.e., the four scales), which are presented as mean and standard deviation as the data were normally distributed. No missing data in valid questionnaire. Second, Pearson’s correlation was used to test the associations between the main study variables. Next, multiple linear regression was performed with job satisfaction as the dependent variable in Model 1 and turnover intention as the dependent variable in Model 2. The independent variables included sociodemographic characteristics, hope, career identity, and job satisfaction (see the Results for details). These statistical analyses were conducted using SPSS version 24.0. The results were considered statistically significant when *p* < 0.05. Finally, AMOS was used to conduct structural equation model (SEM), with turnover intention as the dependent variable, hope and career identity as independent variables, and job satisfaction as the mediating variable to test its mediating effect.

## Results

### Characteristics of the participants

The sociodemographic characteristics of the sample are displayed in Table 1. Almost all of the participants were female (98.6%) and the average age was 31.8 years (SD = 7.4). The majority were 25–44 years of age (75.3%) and married (65.3%). More than half held a Bachelor’s degree or higher (63.5%), and most of them held a junior professional title (71.4%) and no management position (94.3%).

**Table 1:** Sociodemographic characteristics of the sample (*N* = 493)

	*n*	*%*
Age (years)		
< 25	76	15.4
25–44	371	75.3
> 44	46	9.3
Gender		
Female	486	98.6
Male	7	1.4
Marital status		
Single	160	32.5
Married	322	65.3
Others	11	2.2
Education level		
Vocational education	6	1.2
Advanced diploma	174	35.3
Bachelor or higher	313	63.5
Years of work		
< 5	145	29.4
5–19	266	54.0
> 19	82	16.6
Professional title		
Junior	352	71.4
Senior or above	141	28.6
Management position		
None	465	94.3
Officer or above	28	5.7
Work unit		
Surgical	152	30.8
Medical	173	35.1
Women & Child	37	7.5
Others	131	26.6

### Participants’ hope, career identity, job satisfaction and turnover intention

The descriptive statistics for hope, career identity, job satisfaction, and turnover are shown in Table 2. The average score of hope is 22.67 ± 3.57, and paths thinking scored higher than agency thinking (11.77 ± 1.83 vs. 10.90 ± 2.04). The average score of career identity is 110.22 ± 19.16, and the average score of items in sense of self efficiency dimension is the highest. The average score of job satisfaction is 20.71 ± 4.55, and the score of external satisfaction dimension is the highest. The average score of turnover intention is 15.54 ± 3.77, and the average score of items is greater than 2, indicating that the participants’ turnover intention is at a high level.

**Table 2:** Descriptive statistics for the ADHS, NCIS, OJS, and ET

Variables	Number of Items	Mean	SD	Cronbach’s alpha
Hope^a^	8	22.67	3.57	0.857
Agency thinking	4	10.90	2.04	0.779
Pathways thinking	4	11.77	1.83	0.754
Career identity^b^	21	110.22	19.16	0.949
Sense of grasp	3	17.23	2.81	0.884
Sense of consistency	4	21.55	4.44	0.892
Sense of significance	3	15.39	3.27	0.739
Sense of self efficacy	3	17.18	2.80	0.881
Sense of self decision	3	14.59	4.02	0.851
Sense of organization’s influence	2	8.31	2.84	0.814
Sense of patients’ influence	3	15.97	3.30	0.806
Job satisfaction^a^	6	20.71	4.55	0.869
General satisfaction	2	6.86	1.81	0.788
Intrinsic satisfaction	2	6.14	1.89	0.740
Extrinsic satisfaction	2	7.70	1.53	0.751
Turnover intention^c^	6	15.54	3.77	0.827
Possibility to resign from present job	2	4.83	1.61	0.788
Motivation to seek another job	2	4.87	1.63	0.629
Possibility to gained an external job	2	5.84	1.27	0.724

^a^5-point scale with a range of 1–5, ^b^7-point scale with a range of 1–7, ^c^4-point scale with a range of 1–4

### Associations among hope, career identity, job satisfaction, and turnover intention

Pearson’s correlations are shown in Table 3. Hope had a moderate positive correlation with career identity (*r* = 0.494) and job satisfaction (*r* = 0.421). We also found a positive relationship between career identity and job satisfaction (*r* = 0.610). There were negative correlations between turnover intention and hope (*r* = − 0.227), career identity (*r* = − 0.342), and job satisfaction (*r* = − 0.501). It is worth noting that was just small level of correlation between turnover intention and hope, while medium level with career identity.

**Table 3:** Correlations between hope, career identity, job satisfaction, and turnover intention (*N* = 493)

	1	2	3
1 Hope	–	–	–
2 Career identity	0.494^***^	–	–
3 Job satisfaction	0.421^***^	0.610^***^	–
4 Turnover intention	−0.227^***^	− 0.342^***^	−0.501^***^

^***^p < 0.001

Table 4 presents the results of the regression analysis, which further show the relationships between hope, career identity, job satisfaction, and turnover intention. Job satisfaction was the dependent variable in Model 1, and hope, career identity, age, and women & children unit were the independent variables. Model 1 accounted for 42.5% of the variance in nurse’s job satisfaction, and revealed that career identity predicted job satisfaction (β = 0.540) better than hope did (β = 0.159). Model 2 accounted for 32.4% of the variance in nurses’ turnover intention (the dependent variable), with job satisfaction, vocational education, gender, women & children unit, surgical unit, other unit, and marital status (married or not) as independent variables. Model 2 also shows that job satisfaction significantly predicted turnover intention (β = 0.500). Interestingly, the model found age was negatively associated with job satisfaction.

**Table 4:** Regression results

Independent variable	*B*	*β*	*t*	*P*	*R*^*2*^	*F*
Model 1					0.425	44.616^***^
Constant	4.864	–	3.823	< 0.001^***^		
Women & Child Unit	1.578	0.092	2.496	0.013^*^		
Age	−0.085	−0.139	−3.227	0.001^**^		
Career identity	0.128	0.540	13.362	< 0.001^***^		
Hope	0.201	0.159	3.926	< 0.001^***^		
Model 2					0.324	17.608^***^
Constant	31.157	–	12.175	< 0.001^***^		
Married	0.897	0.113	2.353	0.019^*^		
Surgical Unit	0.937	0.115	2.657	0.008^**^		
Women & Child Unit	1.281	0.090	2.211	0.028^*^		
Other Unit	0.884	0.104	2.657	0.018^*^		
Gender	−3.456	−0.109	−2.353	0.005^**^		
Vocational education	−3.364	−0.098	−2.557	0.011^*^		
Job Satisfaction	−0.415	−0.500	−10.068	< 0.001^***^		

Model 1: dependent variable: Job satisfaction

Model 2: dependent variable: Turnover intention

*B* Path coefficient, *β* Standardized path coefficient, *R*^*2*^ Coefficient determination, *t* Path coefficient test statistics (critical ratio), *p* Significance, *F* Test for ANOVA

^*^p < 0.05; ^**^p < 0.01; ^***^p < 0.001

### Mediating effect of job satisfaction on the associations of career identity and hope with turnover intention

**Fig. 2:**
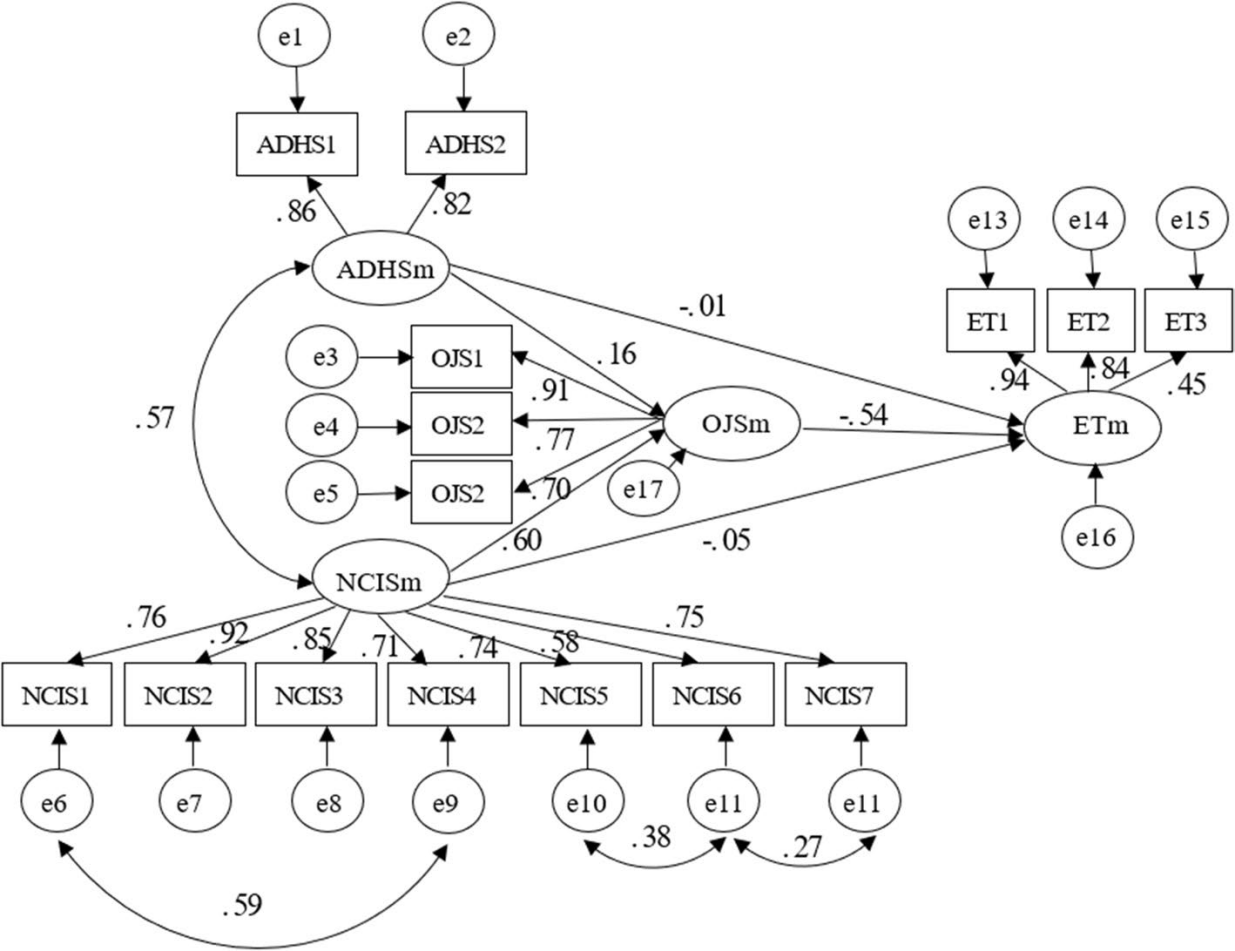
Final model and standardized model paths. ADHSm: Hope; NCISm: career identity; OJSm: job satisfaction; ETm: Turnover; ADHS1: Agency thinking; ADHS2: Pathway thinking; NCIS1: Sense of grasp; NCIS2: Sense of consistency; NCIS3: Sense of significance; NCIS4: Sense of self efficacy; NCIS5: Sense of self decision; NCIS6: Sense of organization’s influence; NCIS7: Sense of patients’ influence; OJS1: General satisfaction; OJS2: Intrinsic satisfaction; OJS3: Extrinsic satisfaction; ET1: possibility to resign from present job; ET2: motivation to seek another job; ET3: possibility to gain an external job

Figure 2 and Table 5 show the SEM results. The final model and standardized model paths are shown in Fig. 2. The total effects, direct effects, and indirect effects are shown in Table 5. The model had a good fit with the data: RMSEA = 0.076, GFI = 0.920, IFI = 0.950, TLI = 0.935, NFI = 0.943, CMIN/DF = 3.865.

**Table 5:** Effects of hope and career identity on turnover intention

Estimate	B	β	SE	95%CI
*Total effect*				
Career identity→Turnover intention	− 0.23	− 0.38	0.06	(− 0.49, − 0.26)
Hope→Turnover intention	− 0.08	− 0.10	0.09	(− 0.23, − 0.03)
*Indirect effect*				
Career identity→Job satisfaction→Turnover intention	−0.21	− 0.33	0.05	(− 0.45, − 0.23)
Hope→Job satisfaction→Turnover intention	− 0.08	− 0.09	0.04	(− 0.17, − 0.03)
*Direct effect*				
Career identity→Turnover intention	− 0.03	− 0.05	0.07	(− 0.18, 0.09)
Hope→Turnover intention	− 0.01	− 0.01	0.06	(− 0.12, 0.11)

A 95% bootstrap confidence interval that does not include zero means an effect is significant

As shown in Table 5, both hope (β = − 0.10, for total effect)) and career identity (β = − 0.38, for total effect) had significant negative relationships with turnover intention. Figure 2 shows there was also a negative relationship between job satisfaction and turnover intention (β = − 0.54). The results also indicated that the indirect effect (β = − 0.09) of hope on turnover intention was significant as well as its direct effect on job satisfaction (β = 0.16). These findings mean that higher hope was related to lower turnover intention and higher job satisfaction, and that job satisfaction completely mediated the relationship between hope and turnover intention.

The results for career identity indicated that it had significant indirect (β = − 0.33) and direct (β = − 0.05) effects on turnover intention as well as its direct effect on job satisfaction (β = 0.60). These findings mean that higher career identity was related to lower turnover intention and higher job satisfaction and that job satisfaction completely mediated the relationship between career identity and turnover intention.

## Discussion

Our study found that hope and career identity were negatively correlated with turnover intention, and that job satisfaction and turnover intention were negatively correlated. Job satisfaction plays a completely mediating role on the associations of hope and career identity with turnover intention.

The average score of the NTIS was 15.54 (SD = 3.77) in this study, and 78.3% of nurses held a strong or extremely strong turnover intention. These results indicate a higher turnover intention compared to those reported in other similar studies. According to Rudman et al. [47], the 5-year cumulative incidence of nurses who strongly intended to leave the profession in Sweden was 30%. In South Korea, the likelihood that new nursing school graduates would leave their first job within 3 years was 46% [48]. The WHO estimated in 2013, that 40% of nurses might leave their work within a decade [49]. The situation in China is also not optimistic. The turnover intention rate of village doctors was 36.8% in 2014 [50], and 45.3% of township health inspectors have been found to have medium to high turnover intention [15]. The mean job satisfaction of nurses in the present study was 20.71 (SD = 4.55), indicating that their job satisfaction was not very high, which is consistent with previous studies [51, 52]. Ghawadra et al., for example, found that 41% of hospital nurses had low job satisfaction [17]. A study of nurses in Turkey found their job satisfaction was at a moderate level [10].

The level of turnover intention of nurses in our study was higher than that reported in many previous studies and their job satisfaction was lower. The reason for this may lie in the nature of our sample, which consisted of registered nurses working in comprehensive tertiary hospitals in Beijing. Comprehensive tertiary hospitals in the Chinese capital have a very large flow of patients who have various diseases that are difficult to treat. Furthermore, the hospitals in our study were all affiliated with a university. Therefore, the nurses had to shoulder the responsibility for teaching nursing students in addition to their regular nursing duties. Thus, it is likely their work was more intensive and stressful, which may lead to lower job satisfaction and high turnover intention. Many studies have demonstrated the effects of high workloads and heavy stress on job satisfaction and turnover intention [2, 5, 17], suggesting that nursing managers should pay more attention to these problems.

Our analyses revealed both career identity and hope were negatively associated with turnover intention. Career identity has a significant association with turnover intention, and it provides a type of intrinsic support. Nurses will devote more energy in and be more enthusiastic about their work when they positively identify with their careers. Career identity may help them to overcome the difficulties and problems that lead to dissatisfaction and strengthen their commitment to stay. Nurses with poor career identity usually tend to leave. Generally speaking, by improving employees’ professional identity, employees’ turnover intention can be effectively reduced. When employees believe that their work can create value and make sense to themselves, they have a higher sense of career identity, more investment and satisfaction with their work; Otherwise, when the career identity is low, employees will consider leaving their existing jobs when appropriate opportunities arise [53, 54]. Furthermore, research has revealed that career identity can influence turnover intention through direct and indirect pathways [8, 15, 18]. Hope and career identity are expressions of intrinsic motivation, which explains why nurses with higher career identity tend to have a positive outlook towards work and retain their hope, thereby having positive effects on many aspects of professional development. A recent quantitative study found widespread pressure on the nurses who helped fight against COVID-19 in Hubei [55]. These nurses mainly had to stay alone in their rooms after work for the sake of safety. The professional identity and hope of nurses were particularly important in this psychological state of pressure and social isolation. Otherwise, this situation would have easily made them think of leaving.

The results of the SEM in our study showed that job satisfaction had a completely mediating effect on the association between career identity and turnover intention. This result is consistent with previous research that job satisfaction had an intermediary role in the relationship between career identity and turnover intention [15]. Job satisfaction is the key incentive variable that determines employees’ turnover intention behavior. In the workplace, employees conduct personal assessment of work characteristics and work environment to generate cognition, emotion and intention, so as to determine their positive or negative evaluation of the nature of work and even the company [56, 57]. This study promotes the development of career identity literature by investigating the intermediary mechanism between career identity and turnover intention. Although early studies have concluded that job satisfaction is a key outcome variable in the process of professional identity [10, 58], previous scholars rarely empirically tested the mediating role of job satisfaction in nurses. Our research shows that job satisfaction is an important way to connect career identity with turnover intention. In addition, we found job satisfaction was an intermediate variable between hope and turnover intention. A previous study showed that psychological capital had a medium to large indirect effect on employees’ turnover intention when hope was part of psychological capital [59]. These results suggest that we can take effective measures to enhance nurses’ hope and career identity so as to improve their job satisfaction and ultimately reduce their turnover intention.

Our study extends prior research results and provides nursing managers with suggestions for team building and management that have theoretical and practical significance. In the face of increasing demands on nurses, the nursing shortage, and proposals to increase the quality of nursing care, nursing managers need to think about how to reduce turnover, retain clinical nurses, and improve the quality of nursing. As discussed above, job satisfaction appears to reduce turnover intention, and to mediate the apparent effects of career identity and hope on turnover intention.

Career identity and hope can affect turnover intention directly and indirectly through job satisfaction. Strengthening career identity and improving hope are critical measures for nursing managers to improve nurses’ job satisfaction, lower turnover intention, and thereby reduce turnover rate and improve the quality of care. Career identity cognitively influences nurses’ attitudes about work. This influence begins when a student enters nursing education and continues throughout his/her working life [18]. Strengthening nurses’ career identity through educational programs is feasible because nurses who did not sufficiently develop career identities during their vocational education can still develop them [18]. In addition, colleges should pay more attention to instilling nursing students with career identity and nursing values [3]. The gap between college and clinical work is one of the reasons for the low level of career identity in newly graduated nurses. Providing them with adequate guidance and support, and enabling them to practice with confidence in a safe and proficient manner may increase their career identity with nursing during this transitional period.

The increased recognition of nurses’ practice is important for expanding the nursing workforce in the future. Hope is influenced by many factors, such as reward, workload, working conditions, doctor-nurse relationships, and management style. Management should increase nurses’ level of hope and job satisfaction in order to retain them by improving their working conditions [17, 60]. This is cost-effective, since training nurses is costly and resources can be directed from being wasted efforts to productive activities [47]. Furthermore, providing nurses with more support, helping them find a spiritual foundation [17], and holding mindful activities that stimulate positive emotions are helpful for improving nurses’ level of hope. Of course, increasing one’s entire psychological capital is beneficial for having hope play a role in professional development.

### Limitations

Our study has some limitations. First, though the sample was large and representative, the generalizability of its results is limited because the sample came from tertiary hospitals in Beijing, all of which were comprehensive teaching hospitals of medical colleges, and thus, these nurses may have characteristics that increase turnover intention. Therefore, further research should be conducted with samples taken from a broader population of nurses (such as nurses from other cities or hospitals of different grades). Second, though the current findings found job satisfaction mediated the association between hope and turnover intention, other variables may contribute to this effect (for instance, social support may influence the relationships among these variables). Future studies should explore other possible mediating variables to reach a more definitive conclusion. Last but not least, because the mediating effect implies causality, it is lack of sufficient persuasion to verify the mediating effect with cross-sectional data. Therefore, the mediating effect explored in this study needs further experimental verification.

## Conclusions

The results of this study support the relationship among hope, career identity, job satisfaction and turnover intention, which guide the intervention measures driven by theory to solve the problem of high turnover intention of nursing staff and help to solve the shortage of nurses. Thus, we can take effective measures to enhance nurses’ hope and career identity in order to improve their job satisfaction and ultimately their turnover intention. Providing nurses with more support, helping them find a spiritual foundation, and holding mindful activities that stimulate positive emotions are helpful. In addition, colleges should pay more attention to instilling nursing students with career identity and nursing values.

## Data Availability

The datasets used and/or analysed during the current study are available from the corresponding author on reasonable request.

## Abbreviations

STROBE statement: Strengthening the Reporting of Observational studies in Epidemiology (STROBE) statement
WHO: World Health Organization
SEM: Structural Equation Model
ADHS: Adult Dispositional Hope Scale
NCIS: Nursing Career Identity Scale
NTIS: Nurse Turnover Intention Scale
SPSS: Statistical Product and Service Solutions
SD: Standard Deviation
COVID-19: Corona Virus Disease-19
